# Buffalo Medical College

**Published:** 1848-02

**Authors:** 


*Buffalo Medical College.*—In the notice of the determination to change the time of lectures in this Institution, and extend the term, which was contained in our last number, a portion of a paragraph was accidentally omitted by the compositor, leaving the reader to infer the sense from the general tenor of the article. To avoid any misapprehension we would repeat that the Faculty have resolved that the session of 1848–9 shall commence on the last Wednesday of November, *and continue five months;* the lecture term being preceded, as now, by a dissecting term of one month, the whole course, including the latter, making six months.

The coming spring session commences on the last Wednesday in February, and continues sixteen weeks. The preliminary dissecting course is now in progress, about thirty students being in attendance.

